# Highlights of the IUBMB education session at the 20^th^ IUPAB congress, 45th Annual SBBf Meeting, and 50th Annual SBBq Meeting

**DOI:** 10.1007/s12551-021-00887-6

**Published:** 2021-11-24

**Authors:** Manuel João Costa, Vera Maria Treis Trindade, Erin Dolan, Luciane V. Mello

**Affiliations:** 1 School of Medicine, University of Minho, Braga, Portugal; 2 Departamento de Bioquímica, Instituto de Ciências Básicas da Saúde, Universidade Federal Do Rio Grande Do Sul, Porto Alegre, Brazil; 3 Department of Biochemistry & Molecular Biology, University of Georgia, Athens, GA 30602 USA; 4 School of Life Sciences, University of Liverpool, Crown Street, Liverpool, L69 7ZB UK

Some significant problems related to undergraduate education in biochemistry and molecular biology magnified by COVID-19 had been recognized previously (AAAS 2011; NRC 2003). Taking place 18 months after the pandemic declaration, this IUBMB education session staged innovative approaches in biochemistry and molecular biology education and provided inspirational examples of research-informed active learning and skill development approaches.

Erin Dolan (University of Georgia, USA) highlighted the power of integrating Course-based Undergraduate Research Experiences (CUREs) into undergraduate course experiences to influence student retention and improve students’ likelihood of graduating from university and majoring in a STEM discipline (Fig. 1). These CUREs involve all students who enroll in a course in addressing research questions of interest to the scientific community. The impact of CUREs was shown with results of the University of Texas’s “Freshman Research Initiative,” involving approximately 1000 students per year (Rodenbusch et al 2016). Providing students with opportunities to make discoveries that build on and contribute to a body of knowledge through iterative work (e.g., problem-solving, troubleshooting, repeating experiments) was found important for CUREs to influence students’ education and career plans (Corwin et al 2018).

**Fig. 1:**
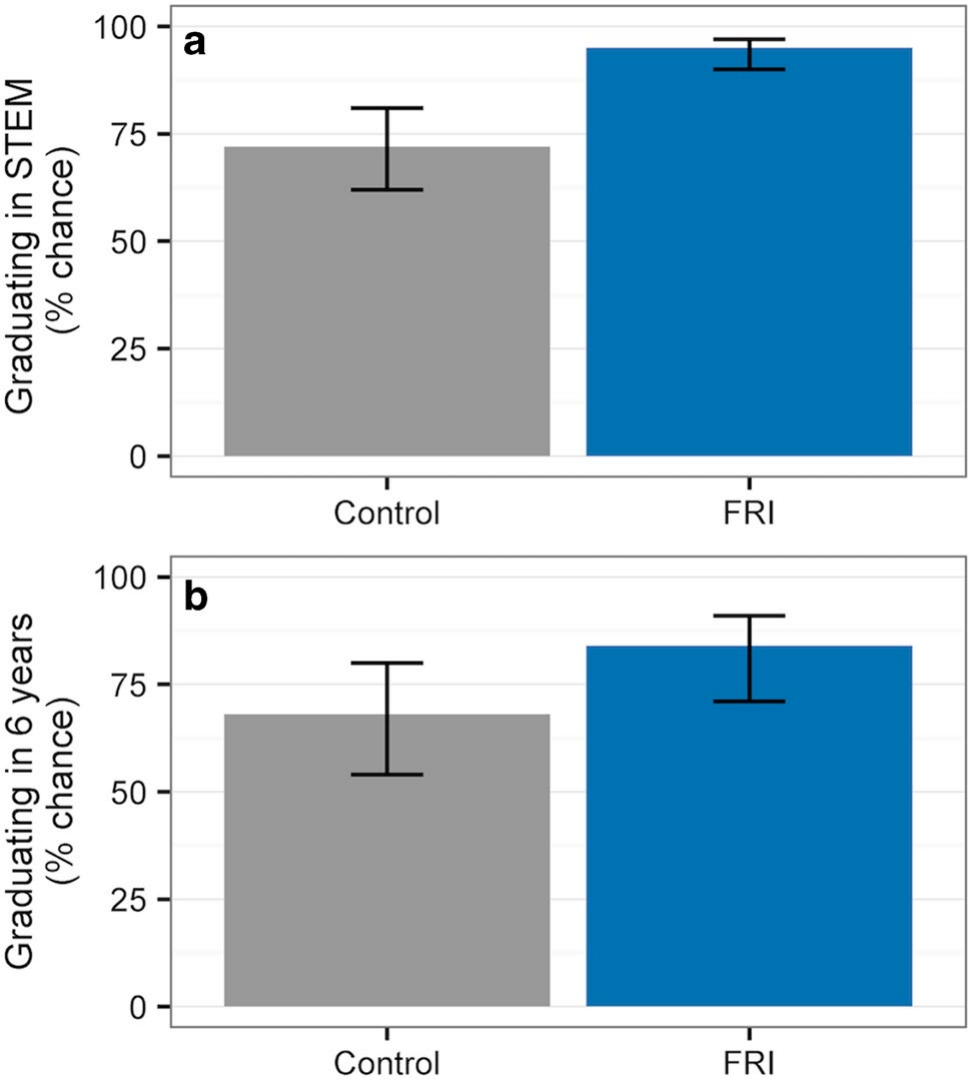
Participation in FRI significantly improves students’ predicted probability of graduating with a STEM major (a) and graduating from the university (b). Error bars represent 98.3% confidence intervals; *p* < 0.017. (Originally published in CBE – *Life sciences Education* under a Creative Commons license, (Rodenbusch et al 2016)

Luciane Vieira de Mello (University of Liverpool, UK) demonstrated the benefits of requiring students to engage in reflective practice to enhance their life and employability skills (Mello et al 2021). Recognizing that students are not always able to reflect on their skills development or on the connection between theory, practice, and their learning, the presentation argued that the science curriculum should allow more reflection and employability skills development (Mello and Wattret 2021). A structured reflective online log summative assessment was introduced. As a result, students acknowledged the importance of self-reflection and appreciated the usefulness of the reflective activity in relation to their future career development.

Manuel João Costa (University of Minho, Portugal) discussed post-pandemic scenarios for learning and teaching. Considering the need to enhance face-to-face education with digital approaches, synchronously and asynchronously, the presentation considered the advantages and disadvantages of each format and established the imperative that new designs are framed under active learning paradigms (Costa and Rangachari 2009; Lino-Neto et al 2021). The power of hybrid approaches to nurturing student engagement and success was illustrated with a year 1 digitally enhanced biochemistry pre-pandemic face-to-face biochemistry course at the University of Minho.

Vera Treis Trindade (Universidade Federal do Rio Grande do Sul, Brazil) presented the “Biokimi App” to support student learning of hepatic glycolysis and gluconeogenesis regulations (Oliveira et al. 2021). It is an android system APP with several screens, divided into 10 modules. The App describes the regulatory steps using images, explanatory texts, and exercises. It is currently available in Portuguese only. Apps such as *Biokimi* are used by students as primary study materials and also as course review interactive resources (Trindade et al 2013).

## References

[CR1] American Association for the Advancement of Science (AAAS). Vision and Change in Undergraduate Biology Education: A Call to Action 2011. https://live-visionandchange.pantheonsite.io/wp-content/uploads/2013/11/aaas-VISchange-web1113.pdf. Accessed 15 Oct 2021

[CR2] Corwin LA, Runyon CR, Ghanem E, Sandy M, Clark G, Palmer GC, Reichler S, Rodenbusch SE, Dolan EL (2018). Effects of discovery, iteration, and collaboration in laboratory courses on undergraduates’ research career intentions fully mediated by student ownership. CBE—Life Sci Educ.

[CR3] Costa MJ, Rangachari PK (2009). The power of interactive teaching. Biochem Mol Biol Educ.

[CR4] Lino-Neto T, Ribeiro E, Rocha M, Costa MJ (2021) Going virtual and going wide: comparing Team-Based Learning in-class versus online and across disciplines. Educ Inf Technol 1–19.10.1007/s10639-021-10683-0 10.1007/s10639-021-10683-0 PMC8366158 34421327

[CR5] Mello LV, Wattret G (2021). Developing transferable skills through embedding reflection in the science curriculum. Biophys Rev.

[CR6] Mello LV, Varga-Atkins T, Edwards S (2021). A structured reflective process supports student awareness of employability skills development in a science placement module. FEBS OpenBio.

[CR7] National Research Council (NRC) (2003). Bio 2010: transforming undergraduate education for future research biologists.

[CR8] Oliveira CH, Ferreira AG, Silva CRCA, Mezalira SM, Trindade VMT (2021) Jogos digitais/analógico, atividades lúdicas & aprendizagem significativa. In: Lima JR, Oliveira MCA, Cardoso (org) Enebio: itinerários de resistência – pluralidade e laicidade no Ensino de Ciências e Biologia, Realize Editora, Campina Grande, pp 678–687

[CR9] Rodenbusch SE, Hernandez PR, Simmons SL, Dolan EL (2016). Early engagement in course-based research increases graduation rates and completion of science, engineering, and mathematics degrees. CBE—Life Sci Educ.

[CR10] Trindade VMT, Zanatta G, Arantes PR, Blanco IDS, Demore FP, Salbego CG (2013). Virtual laboratory activities in basic biochemistry. Procedia Soc Behav Sci.

